# Nanoparticle-Mediated Nucleic Acid Delivery Systems in Plant Biotechnology: Recent Advances and Emerging Challenges

**DOI:** 10.3390/plants14233649

**Published:** 2025-11-29

**Authors:** Tengwei Wang, Jiaxin Li, Ruibin Hu, Xuping Shentu, Zihong Ye, Xiaoping Yu, Kai Sun

**Affiliations:** 1 Key Laboratory of Microbiological Metrology, Measurement & Bio-Product Quality Security, State Administration for Market Regulation, College of Life Science, China Jiliang University, Hangzhou 310018, China; s23090710054@cjlu.edu.cn (T.W.); l1366259702@163.com (J.L.); stxp@cjlu.edu.cn (X.S.); zhye@cjlu.edu.cn (Z.Y.); yxp@cjlu.edu.cn (X.Y.); 2 Zhejiang Provincial Key Laboratory of Biometrology and Inspection & Quarantine, China Jiliang University, Hangzhou 310018, China; 3 Xianghu Laboratory, Hangzhou 311231, China; huruibin@xhlab.ac.cn

**Keywords:** plant transgenesis, nanoparticles, plant genome engineering, nucleic acid delivery

## Abstract

Efficient delivery of exogenous genetic material remains a core challenge in plant biotechnology, holding profound implications for sustainable agricultural and forestry development. Although traditional delivery methods such as *Agrobacterium*-mediated transformation, gene gun bombardment, and electroporation have been widely applied in plant genetic engineering, these systems exhibit limitations including species-dependent efficacy, propensity to cause plant tissue damage, low transformation efficiency, susceptibility to environmental factors. In recent years, with the advancement of nanotechnology, nanoparticle-based nucleic acid delivery systems are emerging as novel tools for applications such as novel tools for dsRNA or transgene delivery. These systems leverage the unique physicochemical properties of nanomaterials, including size-dependent phenomena, tunable surface charge, and enhanced membrane penetration capabilities, to achieve targeted delivery and stable expression of genetic payloads. Nevertheless, nanomaterial-mediated gene delivery systems for plants are still in their nascent stages, and their widespread application faces numerous challenges. This article briefly introduces traditional delivery methods, systematically reviews the applications and progress of nanoparticle-based nucleic acid delivery systems, and discusses the cross-species applicability of nanoparticles, as well as the associated biosafety concerns. We aim to offer insights for tackling the prevailing technical bottlenecks and to provide guidance for the rational design of nanomaterials that efficiently traverse the plant cell wall–plasma membrane barrier and stably deliver nucleic acids without eliciting phytotoxicity.

## 1. Introduction

Contemporary agricultural systems face unprecedented environmental pressures, with synergistic effects of population growth and climate change exacerbating freshwater scarcity, extreme weather events, and soil salinization. These compound abiotic stresses pose critical threats to 21st-century food security and sustainable agriculture [1]. While traditional breeding techniques—including phenotypic selection and mutagenesis—have historically enhanced crop yields and agronomic traits [2], their inherent limitations are increasingly apparent: prolonged breeding cycles (typically 8–10 generations) and reliance on intraspecific genetic diversity hinder the directional transfer of advantageous traits across species [3]. To meet the projected food demands of a global population nearing 10 billion by 2050, plant genetic engineering—through the introduction of exogenous functional genes—has emerged as a pivotal complementary strategy to conventional breeding for rapidly enhancing yield and stress resilience [4].

As a complementary strategy to conventional breeding, plant genetic engineering enables trait modification through the introduction and expression of exogenous functional genes, forming one of the cornerstones of modern molecular breeding, albeit predominantly via random genomic integration. However, despite mature transformation protocols in model plants (e.g., *Arabidopsis*, tobacco), approximately 70% of plant species remain recalcitrant [5,6]. These limitations stem from the multidimensional defense mechanisms of plant cell walls—dynamic three-dimensional structures (0.1–10 μm thickness) composed of cellulose microfibrils, hemicellulose crosslinking networks, and pectin matrices. Beyond their roles in mechanical support and pathogen defense [7], these structures impose strict size exclusion (pore threshold < 20 nm) that restricts passive diffusion of macromolecules. Additional barriers include the selective permeability of plasma membranes, the dual-layer nuclear envelope, and organelle compartmentalization, collectively reducing the transmembrane efficiency of genetic cargoes (e.g., DNA/RNA-protein complexes). To overcome these biological constraints, researchers are developing *Agrobacterium*-independent delivery platforms through multiple approaches. Such interdisciplinary innovations aim to establish more universal genetic modification platforms for crops, though challenges such as genotype-dependent efficiency and cellular barrier penetration remain significant hurdles.

The advent of nanotechnology in the late 20th century has catalyzed the development of diverse functional nanomaterials. Their quantum size effects (<100 nm), high surface-to-volume ratios, and tunable surface chemistry confer unique advantages for nucleic acid delivery [8]. Although these systems have been extensively applied in animal systems for tumor-targeted therapy and gene editing [9], they are now revolutionizing plant science. Engineered lipid-based, metallic, and mesoporous nanocarriers have achieved efficient gene delivery in model plants (e.g., *Arabidopsis* [10], tobacco [11]). This review systematically analyzes the applications of diverse nanomaterials in nucleic acid delivery, highlighting their superior transformation efficiency and species adaptability compared to conventional methods, and elucidating their mechanisms for overcoming cellular barriers. Furthermore, it examines the challenges and prospects of nanomaterial-based delivery systems from the dimensions of toxicity and biosafety, proposing innovative strategies to optimize carrier design principles and provide novel perspectives for the development of next-generation plant genetic engineering platforms.

## 2. Traditional Methods of Plant Transformation

### 2.1. Agrobacterium-Mediated Transformation

Traditional plant genetic transformation involves the artificial introduction of exogenous genes into plant cells for stable expression. The earliest method, *Agrobacterium*-mediated transformation, utilizes *Agrobacterium tumefaciens* or *A. rhizogenes*—Gram-negative soil bacteria naturally capable of infecting dicotyledonous plants through chemotactic specificity, inducing crown gall or hairy root formation via horizontal gene transfer [12]. This system exploits the Ti plasmid transfer DNA (T-DNA) region, into which target genes are inserted and subsequently delivered into plant cells during infection. The T-DNA integrates randomly into the host genome through mechanisms involving virulence proteins [13,14]. Common delivery protocols include vacuum infiltration, floral dip, or direct injection, making this method the gold standard in plant biotechnology due to its operational simplicity and high stability. It has been successfully applied to staple crops such as rice [15], barley [16], wheat [17], and maize [18].

However, critical limitations persist. The system is inherently restricted to DNA delivery and exhibits strong species specificity [19]. Most monocotyledonous plants lack natural susceptibility to *Agrobacterium*, resulting in lower transformation efficiencies compared to dicots. Furthermore, random genomic integration of foreign DNA may disrupt endogenous regulatory elements, potentially leading to unintended phenotypic consequences [20].

### 2.2. Biolistic Particle Delivery (Gene Gun)

Biolistic transformation employs high-pressure helium (typically >1500 psi) to accelerate DNA-coated metallic microparticles (0.6–1.2 μm gold/tungsten) at supersonic speeds (≥450 m/s), enabling penetration through cell walls, plasma membranes, and nuclear envelopes [21]. The delivered DNA integrates randomly into the host genome via non-homologous end joining (NHEJ). Unlike *Agrobacterium*-dependent methods, this technique demonstrates superior performance in monocot transformation systems (e.g., maize embryogenic callus) with efficiencies reaching 15–30%. Optimization strategies involving particle parameters (DNA density: 0.25–1.0 μg/mg gold), bombardment pressure (7.6–28 MPa), and tissue preconditioning (osmotic treatment) have expanded its application to previously inaccessible or difficult-to-transform systems such as certain meristems and somatic embryos [22].

Gene gun technology is renowned for its broad-spectrum transformation capability across species and organelles, enabling simultaneous targeting of nuclear genomes and genetic systems such as chloroplasts. However, its associated drawbacks cannot be overlooked: low and unstable expression efficiency, uncontrollable DNA insertion sites, tissue damage induced by high-pressure vacuum conditions, compounded by substantial DNA requirements, high-precision instrumentation, and elevated costs [23]. These limitations collectively constrain its adoption in both routine experimentation and large-scale applications.

### 2.3. Electroporation

Protoplast electroporation technology (referring to intact plant cells following cell wall removal) was established in in vitro experiments in 1985 [24]. Electroporation utilizes pulsed electric fields (typically 0.5–10 kV/cm) to transiently permeabilize the plasma membrane, thereby facilitating the intracellular delivery of nucleic acids [25]. Its advantages include rapid operation, low cost, and applicability to single cells or cell clusters [26]. However, the method is inherently constrained by its dependence on established protoplast isolation and culture systems; it is incompatible with intact, thick-walled cells. Moreover, supraoptimal field strengths frequently engender irreversible plasma-membrane disruption and attendant cellular dysfunction [27]. Moreover, intense electric field pulses can fragment exogenous nucleic acids, generating truncated or mutated transcripts that may produce non-functional or cytotoxic proteins [28].

### 2.4. PEG-Mediated Delivery

Polyethylene glycol (PEG)-mediated transformation leverages the polymer’s membrane-fusogenic potential under divalent-cation (Mg^2+^/Ca^2+^) conditions to precipitate DNA and facilitate its endocytic uptake [29]. Originally developed for protoplast fusion, the protocol is now predominantly employed for transient gene expression in isolated protoplasts. Its principal advantages include low cytotoxicity, operational simplicity, and mild reaction conditions [30]. The major limitation, however, remains the recalcitrance of protoplast-to-plant regeneration: efficient and reproducible regeneration systems have yet to be established for most plant species [27]. Consequently, PEG-based approaches are constrained by genotype dependency, are applicable only to taxa with defined protoplast culture regimes, and are restricted to isolated protoplasts, being typically utilized for transient rather than stable gene expression [30].

A comparative analysis of traditional delivery methods (Table 1) highlights their inherent trade-offs, such as genotype dependence, tissue damage, and the challenges of plant regeneration from protoplasts (e.g., Electroporation and PEG-mediated transformation). These limitations underscore the need for nanoparticle-based alternatives capable of overcoming these biological barriers.

## 3. Nanoparticles for Nucleic Acid Delivery in Plants

Since the mid-20th century, nanotechnology has been pivotal, catalyzed by Feynman’s 1959 lecture [35]. Nanoparticles (<100 nm) [36] are 0D–3D constructs [37] whose high surface-area-to-volume ratio and tunable chemistry permit precise biomolecular interactions; they are synthesized from carbon, lipid, protein, polymer, ceramic, metal, or metal-oxide matrices [38].

Traditional nanoparticle synthesis is classified into two approaches: (a) top-down, where bulk materials are fragmented into nanoparticles via methods like laser ablation, etching, sputtering, and lithography; and (b) bottom-up, involving the hierarchical assembly from atomic or molecular units using techniques such as hydrothermal/solvothermal methods and co-precipitation [39,40,41].

Based on composition, morphology, size, and origin, nanoparticles (NPs) are primarily classified into three categories: (1) Organic nanoparticles: Composed of lipids, polymers, or proteins (e.g., lipid nanoparticles, micelles, dendrimers, and exosomes). (2) Inorganic nanoparticles: Including metallic nanoparticles (e.g., gold, silver) and metal oxides (e.g., ZnO, Magnetic Nanoparticles) and Silicon-Based Nanomaterials (e.g., Mesoporous silica nanoparticles). (3) Carbon-based nanoparticles: Encompassing carbon nanotubes (CNTs), fullerenes, and graphene. Each category possesses distinct properties and broad applications, continuously driving innovation in nanotechnology and materials science.

**Figure 1:**
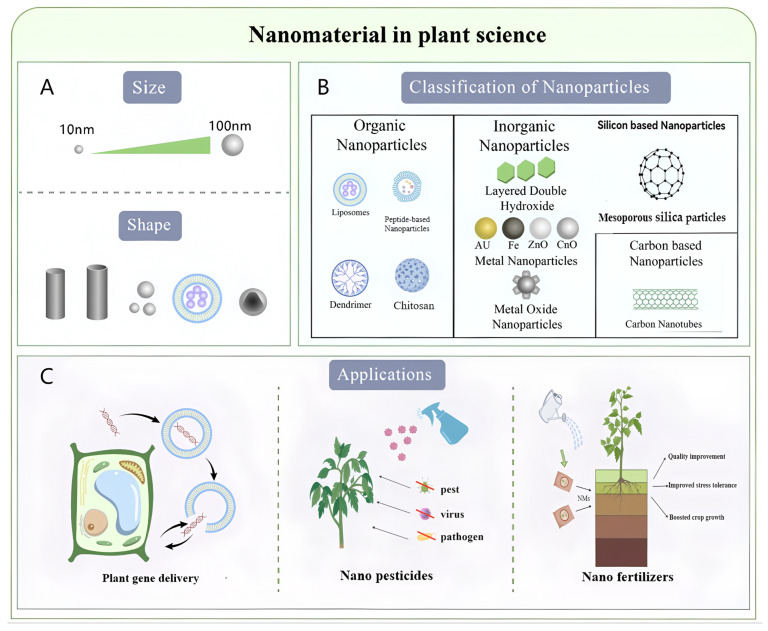
Characteristics, Classification, and Applications of Nanoparticles in Diverse Fields. (**A**) The characteristic parameters of nanoparticles primarily encompass their size and morphological features. (**B**) Nanoparticles can be systematically classified into three major categories: organic nanoparticles, inorganic nanoparticles, and carbon-based nanostructures. (**C**) Current applications demonstrate significant potential in plant gene delivery, nano fertilizers, and nano pesticides, representing cutting-edge implementations across interdisciplinary domains.

Figure 1 schematically illustrates the classification and multifunctional applications of representative nanoparticles in plant biotechnology, highlighting their size-morphology relationships (Figure 1A,B) and interdisciplinary utility in nucleic acid delivery (Figure 1C).

The advancement of nanobiotechnology, which involves the design of nanomaterials for biological systems, has introduced novel dimensions to pharmaceutical development, drug delivery, and interdisciplinary integration with chemistry, biomedical engineering, and materials science. In plant genetic engineering, nanomaterials are increasingly utilized as carriers for delivering plasmid DNA [42], short interfering RNA (siRNA) [43,44,45], and proteins [46] into plant tissues. Beyond genetic engineering, the unique properties of nanoparticles have also paved the way for other groundbreaking applications in agriculture, including the development of nano fertilizers [47] for enhanced nutrient use efficiency, nano pesticides [48] for targeted pest management (as illustrated in Figure 1C).

**Figure 2:**
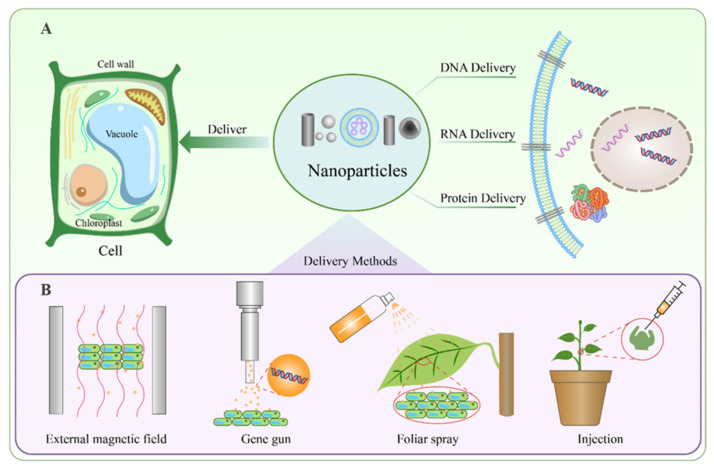
Nanoparticle-Based Delivery Systems for Plant Cells. (**A**) Functions of nanoparticles in plant cells. Nanoparticles can facilitate the delivery of various biomolecules, including DNA, RNA, and proteins, into plant cells. These nanoparticles penetrate the cell wall and membrane to transport genetic material or proteins to specific intracellular locations. (**B**) Methods for nanoparticle delivery in plants. Several techniques can be employed for efficient nanoparticle-mediated delivery, including the use of an external magnetic field, gene gun bombardment, foliar spray application, and direct injection into plant tissues.

Nanoparticle (NP)-mediated biomolecule delivery leverages the unique physicochemical properties of NPs to overcome plant-specific biological barriers. Their small size, high tensile strength, and tunable surface functionalization enable penetration through plant cell walls and tissue matrices. Figure 2 schematically summarizes the principal applications and operational modalities of nanoparticle-mediated delivery: panel A illustrates the capacity of nanoparticles to ferry DNA, RNA, and proteins into plant cells, whereas panel B enumerates implementation strategies—including magnetic-field-assisted and foliar-spray approaches—whose detailed parameters are compiled in Table 2.

### 3.1. Carbon-Based Nanoparticles

Carbon-based NPs, composed primarily of carbon atoms [14], include carbon nanotubes (CNTs), carbon dots (CDs), graphene, graphene oxide, and nanodiamonds.

#### 3.1.1. Carbon Nanotubes (CNTs)

Carbon nanotubes (CNTs) are cylindrical nanostructures formed by rolled graphene sheets [49], exhibiting a tubular morphology with diameters typically ranging from 1 to 2 nm [50]. These nanomaterials demonstrate exceptional mechanical strength, superior electrical conductivity, and remarkable thermal conductivity. CNTs are classified into two primary categories: single-walled carbon nanotubes (SWNTs) [51] and multi-walled carbon nanotubes (MWNTs) [52]. SWNTs generally exhibit diameters < 2 nm, while MWNTs display a broader diameter spectrum spanning 5–100 nm. Notably, both variants can achieve lengths extending from several micrometers to millimeters under optimized synthesis conditions [53,54].

Liu et al. first reported the passive internalization of single-walled carbon nanotubes (SWNTs) into walled plant cells (*Nicotiana tabacum* BY-2) without external assistance, establishing SWNTs as versatile platforms for biomolecule delivery and transgenic plant engineering [55]. Their high aspect ratio, biocompatibility, and large surface area facilitate siRNA and plasmid DNA delivery for gene silencing across diverse plant species [56,57,58].

A critical breakthrough emerged from Wong et al. (2016) [59], who elucidated the lipid exchange envelope penetration (LEEP) model to explain NP uptake mechanisms in plant subcellular compartments. This model identifies particle size as the primary determinant of membrane penetration, with thresholds below which NPs cannot traverse lipid bilayers regardless of zeta potential. Guided by LEEP principles, Kwak et al. [10] engineered chitosan-complexed SWNTs for pH-dependent DNA release in chloroplasts (pH ~ 8), protecting nucleic acids from cytosolic nucleases (pH ~ 5.5) and enabling targeted transgene expression in *Eruca sativa*, *Nasturtium officinale*, and *Spinacia oleracea*.

Further innovations include Santana et al.’s chloroplast-targeted SWNTs functionalized with TOC (translocon outer channel)-binding peptides, which enhanced plasmid delivery efficiency through selective binding to chloroplast membranes [60]. Recent work on German chamomile (*L chamomilla M*) demonstrated polyethyleneimine-coated CNTs as effective ssDNA-FITC carriers, where ultrasonication synergistically enhanced gene transfer rates by generating cavitation-induced pores in cell walls [61].

Owing to the barrier effect imposed by cellulose-rich cell walls on multi-walled carbon nanotubes (MWCNTs), their application is currently confined to protoplast-based delivery systems. In contrast, single-walled carbon nanotubes (SWNTs) have emerged as predominant vectors for nucleic acid transport due to their superior cell wall penetration capacity [62].

#### 3.1.2. Carbon Dots (CDs)

CDs, or carbon quantum dots (discovered in 2004), are fluorescent NPs (1–10 nm) with superior photostability [63,64]. Applications span biomedicine, gene delivery, and bioimaging [65,66,67], leveraging their cellular uptake and translocation efficiency.

Moreover, the regulatory effects of carbon dots (CDs) on plant growth, developmental processes, photosynthetic efficiency, and biotic/abiotic stress resistance have recently emerged as a focus of extensive investigation in phytobiology. Significantly, the successful intracellular delivery of nucleic acid-functionalized CDs through advanced vehiculation strategies have substantially expanded their application spectrum in plant biotechnology, primarily for spray-induced RNA interference and transient nucleic-acid delivery and stress resilience enhancement. Currently, carbon dots (CDs) are recognized as non-toxic alternatives to heavy metal-based quantum dots, and their uptake mechanisms in plant cells have been investigated [68].

Schwartz et al. first demonstrated that polyethyleneimine (PEI)-functionalized carbon dots (CDs) efficiently deliver siRNA into leaves of *Nicotiana benthamiana* and tomato via foliar spraying, triggering systemic silencing of both *GFP* reporter genes and endogenous Mg-chelatase genes [69]. Subsequently, Wang et al. and Delgado-Martín et al. extended this system to dicot (common bean, cucumber) and monocot (rice, wheat) species, successfully delivering plasmid DNA and dsRNA present in cucumber leaves. These studies validated the versatility of PEI-CDs across diverse plant taxa [70,71].

#### 3.1.3. Graphene and Fullerenes

Graphene, a single-layer carbon allotrope featuring a hexagonal honeycomb lattice, exhibits unparalleled electrical conductivity, mechanical strength, and a high surface area. Its derivatives, such as graphene oxide (GO) and graphene quantum dots (GQDs), are of particular interest. GO is a chemically modified graphite derivative characterized by layered architectures of graphene sheets functionalized with oxygen-containing groups, predominantly epoxides, hydroxyls, and carboxylic acids [72]. GQDs represent a class of zero-dimensional carbon-based nanomaterials formed through the dimensional confinement of single- or few-layer graphene into nanoscale fragments (typically <10 nm).

Fullerenes (e.g., buckyballs) are spherical carbon cages composed of pentagonal and hexagonal units linked via sp^2^ hybridization, known for their conductivity, electron affinity, and structural robustness [73].

Although graphene and fullerenes are effective for nucleic acid delivery in mammalian systems [74], their application in plants remains limited due to inefficient internalization and suboptimal delivery efficiency, largely hindered by the plant cell wall and inadequate nanoparticle-plant interactions. For example, while polymer-modified graphene oxide nanosheets can achieve high transient gene silencing (97.2% within 24 h) in plants [75], their capacity for sustained suppression or stable genetic modification requires further investigation. Thus, the utility of graphene and fullerene-based nanomaterials in plant biotechnology remains an open research area.

#### 3.1.4. Post-Graphene

Post-graphene two-dimensional materials denote a family of mono- or few-layer crystals discovered after graphene that possess atomic-scale thickness but deviate from the pure carbon honeycomb lattice.

Owing to their ultrahigh specific surface area, facile surface functionalization, and ability to excite localized surface plasmons or photothermal/photodynamic effects, these post-graphene two-dimensional materials are currently being explored as novel delivery vectors for dsRNA, siRNA, and DNA in plant genetic engineering. Recently, Wei’s team successfully designed a graphitic carbon nitride (g-C_3_N_4_)-based nano-delivery platform and demonstrated that three distinct morphologies of g-C_3_N_4_ can transport exogenous nucleic acids into intact plant cells, albeit with different efficiencies [76]. Among them, carbon-dot-structured g-C_3_N_4_ (g-C_3_N_4_ CDs) exhibited the highest delivery performance and conferred effective protection against Tobacco mosaic virus (TMV) in both *Nicotiana benthamiana* and *Capsicum annuum*. This achievement not only provides a new nano-biological tool for plant gene-function studies and green control of viral diseases, but also highlights the critical influence of morphological architecture on nucleic-acid delivery efficiency, offering a morphological design reference for future plant nano-delivery systems.

### 3.2. Organic Nanoparticles

Organic NPs, such as ferritin, micelles, dendrimers, and liposomes, are biocompatible, biodegradable, and non-toxic. Hollow spherical structures (e.g., micelles and liposomes), also termed nanocapsules, exhibit sensitivity to heat and light [77]. These properties make them ideal for targeted drug delivery systems. Organic/polymeric NPs typically adopt nanosphere or nanocapsule forms [78]. Matrix particles, characterized by solid cores with surface-adsorbed molecules or encapsulated solid masses [79], are widely utilized for controlled release applications.

#### 3.2.1. Peptide-Based Nanoparticles

Peptide-based carriers have emerged as promising non-viral vectors in plant biotechnology [80]. Short cationic amino acid chains electrostatically interact with negatively charged nucleic acids, facilitating cargo encapsulation and delivery [81,82,83]. Functional peptides—including cell-penetrating peptides (CPPs) for crossing cell walls/membranes and organelle-targeting peptides (OTPs) for subcellular localization—enable efficient transport of DNA, RNA, or proteins into plant cells, showing potential for transgenic or gene-edited crop development [84,85].

Early studies revealed histone-derived peptides could traverse petunia protoplast membranes [86], while later work demonstrated CPP-mediated protein delivery into intact tomato and onion tissues [87]. Up to now, CPPs (~30 amino acids) translocate diverse molecules (DNA, proteins, chemicals) into plants, leveraging advantages like cell wall penetration, extracellular stability, and controlled intracellular release [88,89]. Recent advances employ OTPs for organelle-specific delivery (e.g., nucleus, mitochondria, chloroplasts) [90,91]. Thagun et al. fused CPPs with chloroplast-targeting peptides to create peptide-DNA complexes that deliver genetic material into plastids, enabling transient gene regulation without stable transformation [84].

A groundbreaking foliar spray platform developed by Chonprakun Thagun’s group utilizes peptide nanocarriers to deliver bioactive molecules (e.g., siRNA, plasmid DNA) into *Arabidopsis* and soybean (*Glycine max*) cells [92]. Optimization of parameters—including buffer composition—substantially enhanced nucleic acid delivery efficiency. Notably, sprayable peptide carriers enabled targeted siRNA translocation and subsequent knockdown of target genes, offering a non-transgenic route for trait engineering in major crop species. Avital et al. pioneered casein nanoparticle-mediated DNA delivery in *Nicotiana benthamiana* [93]. By modulating CNP zeta potential (pH 4.5), *DsRed*-encoding plasmids were successfully internalized into nuclei, with RT-qPCR confirming gene expression.

Although progress has been made in the delivery of peptide complexes with DNA, dsRNA, etc., critical knowledge gaps remain: (1) The dynamic binding-dissociation equilibrium between the peptide and the genetic cargo under physiological ionic conditions has not been elucidated; (2) The release efficiency of the delivered genetic material is suboptimal; (3) The specific endocytic pathways (clathrin-dependent vs. caveolin-dependent) and the subsequent intracellular trafficking kinetics remain unclear, which ultimately hampers the rational design of peptide-based carriers.

#### 3.2.2. Liposomes

Liposomes are closed spherical structures formed through the self-assembly of at least one lipid bilayer. Their internal aqueous core enables the encapsulation of hydrophilic active agents, while hydrophobic/weakly polar molecules can be embedded within the lipid membrane. Due to their superior biocompatibility and biodegradability, low toxicity, and dual capability to deliver both hydrophilic and lipophilic molecules, these vesicles have become pivotal carriers for efficient transport of small-molecule and macromolecular therapeutics [94,95]. Within this category, cationic liposomes, characterized by their positive surface charge, readily complex with negatively charged nucleic acids such as plasmid DNA and siRNA, rendering them widely utilized vehicles in gene delivery applications.

Nanoliposomes were effective in delivering maize ferredoxin 3 (*ZmFd3*)–dsRNA into corn plants. This delivery silenced endogenous *ZmFd3* gene expression, subsequently conferring significant protection against damage caused by Maize chlorotic mottle virus (MCMV) and/or Sugarcane mosaic virus (SCMV). When applied via foliar spray six days post-viral inoculation, both *ZmFd3*@CLPs and *ZmFd3*@ALPs nanoparticles achieved viral inhibition rates ranging from 52.46% to 79.83% against these pathogens [96].

A lipid-modified polyethylenimine (lmPEI)-based nanoplatform enables the effective silencing of the *RdRp* and *CP* genes in Grapevine leafroll-associated virus 3 (GLRaV-3) through the spray delivery of long double-stranded RNA (dsRNA). This method pioneers a new area in the application of RNAi for managing grapevine viral diseases [97].

Nevertheless, despite the substantial advantages of liposomes as nucleic acid carriers, their complex synthesis procedures hinder the development toward large-scale production and broad applications [95]. No instances of lipid-mediated genetic transformation in intact walled cells have been documented to date.

#### 3.2.3. Exosomes

Exosomes (30–100 nm) are highly biocompatible and robust endosomal-derived extracellular vesicles that serve as efficient nanocarriers for si/miRNA loaded by lipofection, electroporation, or sonication, protecting cargo from RNases and phagocytosis [98,99,100]. Plants exploit exosome-like EVs to export small RNAs (sRNAs) that silence virulence genes in pathogens, exemplified by *Arabidopsis* delivering sRNAs into Botrytis cinerea [101,102]. These vesicles offer low toxicity, small size, innate targeting, and superior barrier penetration, yet their nucleic-acid loading capacity is modest and their plant delivery mechanisms remain unclear [103,104].

#### 3.2.4. Chitosan Nanoparticles

Chitosan is a deacetylated derivative of chitin, primarily extracted from crustaceans, and finds extensive applications in biomedical engineering, agriculture, and food science [105]. Chitosan (CS) exhibits superior dsRNA loading capacity, shields the payload from nuclease degradation and environmental fluctuations in pH and temperature, and enhances foliar adhesion as well as cellular uptake in both plant and insect systems [106].

For instance, chitosan nanoparticles (CS NPs) confer robust protection against dsRNA degradation. Scarpin et al. [107] demonstrated that foliar application of CS–dsRNA complexes effectively suppressed the growth of *Botrytis cinerea*, thereby validating the utility of CS-based RNA interference platforms in plant disease management. Chitosan has also been employed as a foliar-applied carrier for dsRNAs targeting *RsAGO1* and *RsAGO2* of *Rhizoctonia solani*, providing protection against rice sheath blight [108].

Moreover, Petrônio MS and co-workers reported the electrostatic assembly of chitosan-based nanoparticles loaded with dsRNA targeting Tomato mosaic virus (ToMV) to initiate RNA interference-mediated antiviral immunity in plants. Comprehensive physicochemical characterization confirmed particle formation, colloidal stability, and RNA–matrix interactions under optimized stoichiometry, while in vitro toxicological assays demonstrated negligible phytotoxicity and hemolytic activity against human erythrocytes. This study establishes a safe and efficient nanocarrier platform, offering a promising green-strategy for crop protection via RNAi-based pathogen control [109].

Notably, plant cell transformation efficiency is governed by a combination of factors, including chitosan properties (molecular weight and degree of deacetylation), medium pH, and cell type [110].

### 3.3. Inorganic Nanoparticles

Devoid of carbon, inorganic nanoparticles exhibit superior stability, biocompatibility, and hydrophilicity relative to their organic counterparts, and are classified into three main groups: metallic nanoparticles, metal-oxide nanoparticles, and silicon-based nanomaterials.

Metal Nanoparticles: Metal NPs (1–100 nm) exhibit unique physicochemical and optical properties due to localized surface plasmon resonance (LSPR). Their high surface-area-to-volume ratio enhances reactivity, while quantum effects alter electronic and optical behaviors compared to bulk materials. Synthesis is governed by precise control of shape, crystallographic facets, and size [111], with diverse metals (e.g., Au, Ag) forming NPs [112].

Metal Oxide Nanoparticles: Composed of metal-oxygen bonds, these NPs combine metallic and oxide properties. Oxidation of metal NPs (e.g., conversion of Fe to Fe oxide) enhances reactivity. Commonly studied variants include ZnO, SiO_2_, Fe oxides, Al_2_O_3_, CeO_2_, TiO_2_, and magnetite. Furthermore, owing to their reduced dimensions, metal oxide nanoparticles exhibit a superior capacity to penetrate and interact with diverse cellular structures compared to their bulk counterparts. More significantly, these nanoscale particles demonstrate negligible induction of systemic toxicity, which is principally attributed to their significantly enhanced biocompatibility profile [113,114].

#### 3.3.1. Gold Nanoparticles

Early studies by Christou et al. (1990) demonstrated the use of gold microparticles coated with DNA for gene delivery into soybean seeds via biolistic methods [115]. Gold nanoparticles (AuNPs) have since gained prominence in plant science due to their adjustable size/shape, ease of synthesis, versatile surface modification, and optical properties [116,117]. However, their application as standalone gene carriers in walled plant cells remains limited due to challenges posed by the rigid cell wall and poor cargo-loading capacity [27]. Consequently, AuNPs are predominantly employed in bioimaging and as stimuli-responsive cargo release triggers [11].

Recent advances by Huan Zhang et al. highlighted the role of NP morphology in plant gene delivery. A library of DNA-functionalized AuNPs (5–20 nm) with spherical or rod-shaped configurations was evaluated for siRNA transport in mature plant leaves. Smaller spherical AuNPs (10 nm) exhibited faster cell wall binding kinetics, achieving superior siRNA delivery and gene silencing efficiency, with a cell wall size exclusion limit of ~20 nm [118]. Importantly, effective siRNA delivery was achieved in the absence of detectable nanoparticle internalization, underscoring the critical role of surface-mediated interactions. In a preceding study, Zhang and co-workers synthesized ultra-small polyethylenimine-functionalized gold nanoclusters (PEI-AuNCs, 1–2 nm) bearing PEI with molecular weights of 800, 2.5 k and 25 kg/mol; these constructs penetrated the cell wall via electrostatic adsorption of siRNA and mediated robust gene silencing in *Nicotiana benthamiana* [119].

Recent work by Qi’s team demonstrates the rational design of polyethylenimine-functionalized gold nanoparticles (PEI-AuNPs) integrating dual functionalities: fluorescence-enabled tracking and ROS-scavenging activity, which collectively establish a multifunctional siRNA delivery platform. This nanocarrier system achieved 71% silencing efficiency of the *ATWRKY1* gene in *Arabidopsis thaliana*, concomitant with significantly enhanced disease resistance phenotypes in planta, provides a new strategy for breeding to improve disease resistance [120].

#### 3.3.2. Layered Double Hydroxide

Layered double hydroxides (LDHs), a class of inorganic materials with alternating cationic metal hydroxide layers and anionic interlayers, exhibit unique ion-exchange and adsorption properties due to their layered structure. Naturally occurring as minerals, LDHs have found diverse applications in drug delivery, catalysis, energy storage, and environmental remediation [121,122,123,124,125]. In plant biotechnology, LDHs have emerged as promising nanocarriers for nucleic acid delivery owing to their ion exchange and adsorption properties. LDH nanosheets are positively charged lamellar inorganic materials with the general formula [M^2+^_1−x_M^3+^_x_(OH)_2_] [A^n−^]_x/n_·zH_2_O [126].

The delivery mechanisms of DNA/RNA-LDH complexes into plant cells involve three potential pathways: (1) free nucleic acids bypassing cell wall barriers while LDH complexes are excluded, (2) non-endocytic transmembrane transport of complexes, or [14] endocytosis-mediated internalization. A landmark study by Bao et al. demonstrated the utility of LDH-lactate nanosheets (30–60 nm diameter, 0.5–2 nm thickness) for efficient cytoplasmic delivery of fluorescent dyes and DNA in intact plant cells. The neutralized LDH-nucleic acid conjugates showed no cytotoxicity and enabled nuclear localization of ssDNA-FITC, highlighting LDH’s potential as a gene carrier [127].

Recent advancements expanded LDH applications to RNA interference (RNAi) delivery. Mitter’s team developed biodegradable “BioClay” nanosheets that electrostatically bind dsRNA, protecting it from enzymatic degradation while enabling sustained release through CO_2_- and humidity-driven decomposition. When applied to *Arabidopsis* leaves, these dsRNA-LDH complexes triggered systemic antiviral resistance via RNAi, marking a significant leap in plant protection strategies [128]. Further validation in crop systems showed that hexagonal LDH nanosheets (30–90 nm) loaded with dsRNA targeting *Fusarium oxysporum* genes could confer 60-day protection against root rot in tomatoes through foliar, petiole, or root delivery methods [129].

Size-dependent uptake mechanisms were elucidated by Xu’s team, who identified a ~50 nm penetration threshold for LDH/dsRNA nanoparticles in tomato pollen cells. Subsequent studies with 40 nm particles in *Nicotiana benthamiana* revealed extracellular localization, vascular system transport, and chloroplast-targeted delivery facilitated by charge complementarity between cationic LDHs and anionic organelle membranes. Notably, LDH encapsulation preserved payload integrity, even negatively charged cargoes can be transported into cells together with LDH, as demonstrated by pH-sensitive FITC fluorescence retention post-delivery [130,131].

Furthermore, (MgFe)–LDH nanosheets loaded with *SsAgo2*-targeted dsRNA have been successfully employed in spray-induced gene silencing (SIGS) to combat *Streptococcus* species [132]. Notably, the injection of LDH-dsRNAs targeting *MeLRRs* resulted in a significant reduction in cassava resistance against cassava bacterial blight (*Xanthomonas axonopodis* pv. *manihotis*), suggesting that LDH nanosheet-mediated gene silencing may hold broad applicability in agricultural systems [133]. Collectively, LDH nanosheets exhibit promising potential as versatile vectors for plant genetic delivery and are anticipated to be strategically leveraged in the genetic-level prevention and management of diverse phytopathogenic diseases [62].

The environmental benefits of LDH platforms stem from their gradual degradation into biocompatible components under atmospheric conditions. Key advantages include prolonged nucleic acid protection on leaf surfaces, controlled release kinetics, and cross-species applicability. However, critical knowledge gaps persist regarding species-specific leaf surface interactions, intracellular trafficking mechanisms, and long-term ecological impacts of degradation byproducts. Future research should prioritize optimizing LDH formulations for precision delivery while advancing fundamental understanding of plant-nanoparticle interfacial dynamics to fully harness this technology in sustainable agriculture.

#### 3.3.3. Magnetic Nanoparticles (MNPs)

Magnetofection refers to nucleic acid delivery under magnetic forces. Magnetic nanoparticles (MNPs), typically iron oxide coated with biomolecules, serve as efficient gene delivery vehicles that can be directed to target cells via external magnetic fields [134]. First applied for drug delivery in the 1970s and mammalian cell transfection in 2000 [135], this method enables nuclear gene transfer through magnetic guidance [136].

While MNP-based magnetofection has been predominantly explored in animal systems [137], limited studies exist in plant science. A notable breakthrough demonstrated transgenic seed production without tissue culture regeneration via MNP-mediated delivery [138]. By infiltrating cotton pollen grains with *GUS* reporter gene-MNP complexes under magnetic force, researchers generated transgenic plants with successful genomic integration, functional expression, and stable inheritance of exogenous DNA across self-pollinated progeny. The methodology pioneered by Zhao et al. demonstrates the feasibility of delivering exogenous DNA into plant genomes and generating transgenic plants bypassing tissue culture steps traditionally required in plant genetic engineering. However, the broad applicability of pollen magnetofection across diverse plant species and the standardization of species-specific operational protocols necessitate further validation through systematic transformation experiments.

In a recent study, Wang et al. employed MNPs conjugated with enhanced green fluorescent protein (*EGFP*) or bialaphos resistance (*Bar*) genes to transform five maize varieties [139]. *EGFP* fluorescence was observed in 92% of pollen grains and 70% of embryos, while PCR detected the transgene in 29–68% of T1 individuals, of which 7–16% produced immunoreactive *EGFP*. Among *Bar*-transformed T1 lines, 1.41% survived glufosinate selection and were confirmed by Southern blot. The protocol is genotype-agnostic and allows high-throughput DNA introduction into elite maize, offering a practical tool for rapid germplasm engineering.

#### 3.3.4. Silicon-Based Nanomaterials

Silicon-based nanocarriers are widely utilized for gene and drug delivery owing to their chemical inertness and excellent biocompatibility. The abundant surface silanol groups enable facile functionalization, further enhancing both the stability and biological performance of these materials [140]. Among them, mesoporous silica particles—featuring tunable dimensions, low cytotoxicity, high loading capacity, and optical transparency—have emerged as the preferred platform for gene delivery within the silicon nanomaterial family [141]. Martin-Ortigosa et al. [142] further demonstrated that 10 nm large-pore gold-coated MSNs enable co-delivery of pDNA and proteins into *Allium cepa* epidermis; however, the requirement for costly biolistic bombardment to breach the cell wall constrains widespread adoption. A recent study prepared ~40 nm APTES-functionalized MSNs that delivered pDNA-*GUS* into tomato leaves and shoots via simple spraying or micro-injection [143]; the same platform was further employed to introduce the insecticidal *cry1Ab* gene. These findings demonstrate that MSNs can mediate DNA transfer into plant tissues without external assistance, offering a new avenue for optimizing their role as efficient gene vectors.

Cai et al. [144] reported that MSNs achieve transient siRNA-mediated gene silencing in mature plant leaves with an efficiency approaching 98%. This approach enables high-efficiency foliar uptake of siRNA in *Nicotiana benthamiana* without mechanical assistance, achieving 98% silencing of the *GFP* transgene at the molecular level. Lv et al. [145] demonstrated that foliar spraying of rough-walled hollow mesoporous silica (RHMS) enables simultaneous delivery of *CYP6CY13* dsRNA and imidacloprid for synergistic control of *Aphis gossypii*. The hollow mesoporous architecture efficiently accommodates the insecticide, while surface electrostatic interactions condense dsRNA into a nanocomplex that markedly protects the RNA from nuclease degradation.

**Table 2:** Examples of Nanoparticle-Mediated Delivery Systems in Plants.

Nanomaterial	Size Range	Cargo Delivered	Delivery Approach	Plant Species	Transformation Type	Year	References
Gold nanoparticles	5–20 nm	siRNA	Biolistic delivery	*Nicotiana benthamiana*	Transient	2021	[118]
Gold nanoclusters	5–20 nm	siRNA	Leaf infiltration	*Nicotiana benthamiana*	Transient	2021	[119]
PEI—AuNPs	7–8 nm	siRNA	Leaf infiltration	*Arabidopsis thaliana*	Transient	2024	[120]
Chitosan-CNT hybrids	90–120 nm	pDNA	Carrier-free delivery	*Nicotiana tabacum*, *Spinacia oleracea*	Transient	2019	[10]
Cationic carbon nanotubes	100–200 nm	ssDNA	Ultrasound-assisted	*Matricaria chamomilla*	Transient	2020	[61]
Magnetic nanoparticles	140.6–168 nm	DNA	Magnetic field-assisted	*Gossypium hirsutum*, *Zea mays*	Stable	2017, 2022	[138,139]
Peptide carriers	54–77 nm	pDNA/siRNA	Foliar spraying	*Arabidopsis thaliana*, *Glycine max*	Transient	2022	[92]
Casein nanoparticles	81–246 nm	DNA	Electrostatic interaction	*N. benthamiana leaves*	Transient	2024	[93]
Layered double hydroxides	40–45 nm	dsRNA/siRNA	Spraying/leaf infiltration	Tobacco species	Stable/Transient	2017, 2022	[128,131]
Lipid nanoparticles	385 nm	dsRNA	Spraying	Corn	Stable	2023	[146]
Exosomes	30–100 nm	RNA	Biolistic delivery	*Arabidopsis*	Stable	2018	[101]
Graphene	1–100 nm	SiRNA	Internalize	*Nicotiana benthamiana*	Transient	2022	[75]
Mesoporous silica nanoparticles	10 nm	DNA/Protein	Gene gun	*Allium cepa*	Transient	2012	[142]
Mesoporous silica nanoparticles	40 nm	siRNA	Spraying	*Nicotiana benthamiana*	Transient	2024	[144]
G-C3N4	2 nm	dsRNA	Spraying	*Nicotiana benthamiana*	Stable	2025	[76]

## 4. Mechanisms of Nanoparticle Delivery in Plants

The absorption and transport of NPs in plants are influenced by multiple factors, including plant species, particle concentration, size, surface charge, and exposure duration [147]. NPs primarily enter plants through stomata, root hairs, and surface cracks on leaves. Once internalized, their systemic movement relies on diffusion, cytoplasmic streaming, and phloem loading, modulated by particle size, surface properties, solution pH, and coexisting ions or compounds [148].

### 4.1. Foliar Uptake Pathways

The leaf epidermis, protected by a cuticular layer of waxes and cutin, serves as a natural barrier against NP penetration [149,150]. Recent studies using confocal fluorescence microscopy demonstrated that carbon quantum dots < 2 nm in diameter can traverse the cotton leaf epidermis via the cuticular pathway (diffusion through cuticular waxes and cutin matrix) [151]. However, the limited pore size in the cuticle restricts this route. Alternatively, NPs may enter through stomatal apertures, which vary in density and activity across plant species and environmental conditions [152]. For instance, metal-based NPs < 50 nm in diameter efficiently utilize stomatal pathways [153]. Zhu et al. observed that reduced stomatal aperture size decreased zinc oxide NP accumulation in wheat chloroplasts and cytoplasm by 33.2% and 8.3%, respectively [154]. In summary, nanoparticles are predominantly applied to roots or vegetative organs, with leaves representing the preferred route. On foliar surfaces, they passively penetrate via natural apertures—stomata, hydathodes, stigmata, and micrometer-scale bark fissures—whose pore diameters span the nano- to micrometer range [155,156].

After entering mesophyll cells, NPs undergo long-distance transport via apoplastic or symplastic pathways [157]. The apoplastic pathway proceeds through extracellular spaces—including cell walls, intercellular voids, and xylem conduits—and is governed by particle size and surface charge [158]. In contrast, the symplastic pathway relies on plasmodesmata (2–20 nm diameter) for cytoplasmic transport to the endodermis and Casparian strip [159]. Studies suggest particles < 50 nm preferentially utilize symplastic transport, while larger NPs (50–200 nm) favor apoplastic routes [160]. Subsequent translocation from leaves to roots occurs via phloem-mediated vascular transport.

### 4.2. Root Uptake and Transport

Root hair cells absorb NPs, which then traverse cell walls through apoplastic or symplastic pathways to reach the endodermis and xylem vessels [161]. In the apoplastic route, NPs encounter the Casparian strip—a lignified barrier in the endodermis—leading to partial NP deposition or lateral redirection. However, incomplete Casparian strip development at lateral root junctions permits vascular entry [162]. The symplastic route involves NP internalization via plasma membranes and intercellular transport through plasmodesmata. Notably, emerging evidence suggests NPs can transport cargo without cellular internalization [163].

### 4.3. Cellular Uptake Mechanisms

Although endocytosis [164] remains the dominant route for subsequent cellular uptake, alternative entry mechanisms—such as pore formation, direct membrane translocation, or carrier-mediated transport—documented in mammalian cells [165,166,167] and invertebrate [168] models await validation in plant systems. Multi-walled carbon nanotubes, for example, have been shown to escape endosomes during internalization into *Catharanthus roseus* protoplasts [169]. Notably, some nanocarriers, including certain LDH formulations, may not necessarily internalize but instead release their nucleic acid cargo at the cell surface or within apoplastic spaces through ion exchange or dissolution mechanisms [130,131]. Once in the cytosol, intercellular movement of nanoparticles (NPs) is mediated by plasmodesmata—membrane-lined cytoplasmic bridges with a dynamically regulated diameter of 20–50 nm that maintain continuity of both membrane systems and cytosol within plant tissues. Transport of NPs of various sizes through plasmodesmata has been documented in *Arabidopsis*, rice, and poplar [170,171,172].

### 4.4. Research Frontiers and Implications

**Figure 3:**
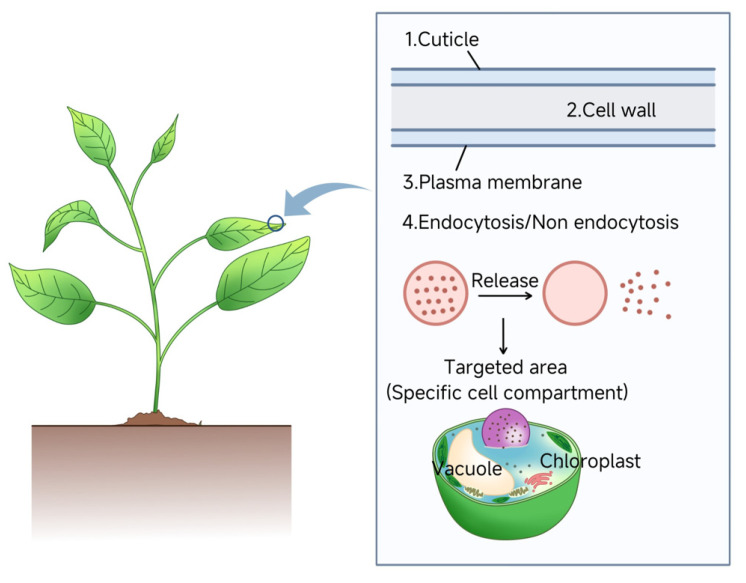
Influence of Cellular Barriers of Nanoparticle-mediated nucleic acid delivery. The efficiency of nanoparticle-mediated nucleic acid delivery is governed by plant-specific cellular barriers. To achieve successful intracellular delivery, nanoparticles sequentially traverse the cuticle, cell wall, and plasma membrane. Internalization occurs via endocytic or non-endocytic pathways.

Current models emphasize NP aspect ratio and rigidity as critical factors for cell wall penetration [173,174]. Interspecies variations in plant morphology and physiology further modulate NP uptake efficiency [175]. Future research should integrate advanced imaging and computational models to decode NP-plant interactions at cellular and subcellular levels. Elucidating these mechanisms will optimize NP-based agricultural technologies and plant genetic engineering applications. Figure 3 illustrates the sequential barriers—that nanoparticles must overcome during cellular uptake after entering plant tissues, providing a framework for advancing the delivery systems listed in Table 2.

## 5. Limitations of Nanoparticles

Although nanoparticles demonstrate potential for growth regulation, sensing, and gene delivery in plant systems, nanoparticle-mediated genetic transformation remains constrained by multiple factors.

### 5.1. Nanoparticle Phytotoxicity

Plant genetic engineering enables the precise and efficient targeted modification of key crop traits, overcoming genetic bottlenecks inherent to conventional breeding. This approach achieves synergistic improvements in both quality and yield, thereby addressing escalating demands for food and industrial applications. However, as emerging delivery platforms, nanocarriers may elicit ecological risks and health implications due to their dimensional effects and surface reactivity—properties that enhance transformation efficiency while simultaneously constituting a core obstacle limiting their scalable implementation.

For instance, carbon nanotubes exhibit carcinogenicity and can induce reproductive and developmental toxicological effects, while also demonstrating environmental persistence due to slow degradation [176]. Conversely, chitosan nanocarriers—both when loaded with ds*GFP* and in their unloaded state—significantly suppress myosin expression in *Caenorhabditis elegans* [177]. Previous studies suggest that nanoparticles can trigger ROS overaccumulation and disrupt hormonal homeostasis in plants, consequently inhibiting plant growth and development [178,179]. Concurrently, nanoparticles may suppress the expression of genes associated with pathogen responsiveness, hormone stimulation, and stress responses, adversely affecting plant defense mechanisms and root development [180]. There is an urgent need to develop novel nanomaterials with reduced toxicity and enhanced delivery efficiency, which can be adapted into nucleic acid delivery platforms. Such platforms should be designed to overcome species-specific biological barriers, including plant cell wall penetration, to significantly enhance genetic transformation efficiency and efficacy across diverse plant species.

### 5.2. Nanoparticle Accumulation

During nanocarrier-mediated genetic transformation, nanocarrier-plant interactions facilitate their trophic transfer along food chains. Consequently, their absorption kinetics and degradation behavior have become critical determinants for safeguarding food security. Furthermore, carrier delivery systems must be engineered to effectively suppress aberrant nanoparticle accumulation in non-target tissues or organs [181].

However, the mechanisms underlying nanoparticle internalization in plant cells and their interactions with intracellular components remain to be systematically elucidated. Furthermore, transmembrane uptake routes, encountered biochemical and physical barriers, intracellular trafficking pathways, and accumulation sites are poorly characterized [182]. Further mechanistic investigations remain to be conducted.

## 6. Summary and Outlook

Compared to conventional transformation methods, nanoparticle (NP)-based delivery platforms demonstrate distinct advantages and broad prospects in plant genetic engineering. NPs not only efficiently transport genetic materials but also deliver diverse bioactive molecules into plant cells. These systems passively penetrate cell walls without external force, enable high-capacity loading of long-chain nucleic acids, and support multigene co-transformation. Under NP protection, exogenous nucleic acids exhibit enhanced stability with significantly reduced intracellular degradation risks, thereby improving transformation efficiency [27]. Surface functionalization and structural engineering of nanoparticles enhance the precision of nucleic acid delivery routes and enable the fabrication of stimulus-responsive controlled-release systems, thereby bolstering plant defense against phytopathogens and herbivorous pests. Furthermore, certain nanomaterials intrinsically possess growth-regulatory and stress-tolerance-enhancing functions, generating synergistic effects. Leveraging nanostructures such as mesoporous frameworks enables integration of multiple active components—including plant growth regulators, fungicides, antivirals, and insecticides—into unified platforms for systemic mitigation of biotic and abiotic stresses during crop growth [62]. Crucially, specific NP-mediated strategies accomplish delivery of exogenous DNA or siRNA to chloroplasts, mitochondria, and other subcellular compartments without requiring chemical induction or biolistic assistance.

Despite significant advances in subcellular delivery, nanoparticle-mediated transfer continues to face substantial challenges. Current systems remain largely dependent on conventional transformation methods such as gene guns or electromagnetic fields, with efficiencies that lag behind microbe-mediated approaches. The plant cell wall continues to represent a formidable barrier, demanding further optimization of nanocarrier design, while the uptake kinetics, translocation pathways, and intracellular interactions of nanoparticles remain insufficiently understood. Moreover, most NP-based strategies yield only transient expression, limiting their cross-species applicability and highlighting the need for regeneration protocols that ensure genetic stability. Cytotoxicity also poses a concern, emphasizing the importance of developing carriers that combine high payload capacity with strong biocompatibility. Collectively, overcoming these barriers is essential to fully unlock the translational potential of nanoparticle-mediated gene delivery in plant biotechnology.

Future research must transcend disciplinary boundaries to integrate carrier design, delivery strategies, plant regeneration, and ecological safety within a unified framework. This integrated approach will establish a “precision-, efficiency-, and sustainability-driven” paradigm for plant genetic engineering, addressing frontier demands in plant science and agricultural biotechnology.

## Figures and Tables

**Table 1:** Comparison of Gene Delivery Methods.

Delivery Method	Target Type	Delivery Material	Advantages	Limitations	References
*Agrobacterium*-mediated	Root, shoot apical meristem, leaf, flower, hypocotyl, cotyledon	DNA	High stability, simplicity, efficiency	Host species limitation, genomic disruption	[31,32]
Biolistic (Gene Gun)	Callus, protoplasts, explants	DNA	Species-independent, simple operation	Low integration efficiency, tissue damage, low expression	[27,28]
Electroporation	Protoplast	DNA	Rapid, efficient, low cost	Cell wall penetration difficulty, tissue damage, Requires protoplast regeneration	[25]
PEG-mediated	Protoplasts	DNA	Low cost, simple operation	Genotype dependency, cellular stress, Requires protoplast regeneration	[29,33]
Nanomaterial-based	Leaves, protoplasts	DNA/RNA/proteins	High versatility, biocompatibility	Complex synthesis, efficiency depen dent on NP properties	[34]

## Data Availability

No new data were created or analyzed in this study. Data sharing is not applicable to this article.
